# Draft Genome Sequence of *Brevundimonas* sp. Strain T2.26MG-97, Isolated from a Rock Core Sample from 492.6 Meters Deep on the Subsurface of the Iberian Pyrite Belt

**DOI:** 10.1128/MRA.00375-19

**Published:** 2019-08-08

**Authors:** E. Rodríguez-Robles, J. M. Martínez, T. Leandro, R. Amils

**Affiliations:** a Centro de Biología Molecular Severo Ochoa (CBMSO, CSIC-UAM), Universidad Autónoma de Madrid, Cantoblanco, Madrid, Spain; b Centre for Neuroscience and Cell Biology, University of Coimbra, Coimbra, Portugal; c Centro de Astrobiología (CAB, CSIC-INTA), Carretera de Torrejón a Ajalvir, Torrejón de Ardoz, Madrid, Spain; University of Maryland School of Medicine

## Abstract

The draft genome of *Brevundimonas* sp. strain T2.26MG-97, isolated at a depth of 492.6 m in the subsurface of the Iberian Pyrite Belt, is reported here. It consists of 262 scaffolds with a total genome length of 3.68 Mbp, where 3,549 coding DNA sequences have been annotated.

## ANNOUNCEMENT

The genus Brevundimonas is classified within the class Alphaproteobacteria and family Caulobacteraceae. *Brevundimonas* species are characterized as Gram-negative, aerobic or facultative anaerobic, rod-shaped, non-spore-forming bacteria (1) and have been isolated from diverse environments. These include extreme environments of astrobiological interest like the Antarctic Dry Valleys (2), Everest (3), ice glaciers in Greenland (4), and the Arctic (5).

*Brevundimonas* sp. strain T2.26MG-97 was isolated under strict anaerobic conditions from a 492.6-m-below-surface rock core sample from the Iberian Pyrite Belt (IPB), in the framework of the Iberian Pyritic Belt Subsurface Life (IPBSL) drilling project (6). The aim of this project was to characterize the subsurface microbial diversity in the IPB, a geological formation in southwest Spain considered a terrestrial geochemical and mineralogical Mars analogue (7). Drilling and sampling were performed as described by Puente-Sánchez et al. (8). Culturing, isolation, and taxonomic identification of *Brevundimonas* sp. strain T2.26MG-97 were carried out as described by Leandro et al. (9).

For whole-genome sequencing, genomic DNA was extracted by the cetyltrimethylammonium bromide (CTAB)-based method (10), and its concentration was determined with a Qubit v.2.0 fluorometer (Invitrogen, USA). It was submitted to the MicrobesNG sequencing facility (University of Birmingham, UK) for Illumina MiSeq sequencing with 27× coverage. A Nextera XT kit (Illumina) was used to prepare the genomic libraries following the manufacturer’s protocol. The run resulted in 242,736 2 × 250-bp paired-end reads with a length between 36 and 251 bp. The reads were trimmed by Trimmomatic v.0.36 (11), and quality analyses of the reads were performed using FastQC v.0.11.8 software (http://www.bioinformatics.babraham.ac.uk/projects/fastqc/). *De novo* assembly was carried out using SPAdes v.3.12.0 (“-careful” option) (12), and the assembly of extrachromosomal genetic elements was performed using Recycler, which uses the read coverage of contigs to distinguish between plasmids and chromosomes (13). Plasmid contigs were aligned against the chromosomal assembly with Mauve Aligner v.2.4.0 (14) to separate chromosomal and plasmid contigs. Contigs were extended and merged into scaffolds using SSPACE software (15). Gaps created with SSPACE were closed with GapFiller v.1-10 software (16). This resulted in a chromosome in 262 scaffolds, with an *N*_50_ value of 35.42 kb, a GC content of 67.20%, and a total size of 3,683,793 bp, and one plasmid of 19,563 bp in two contigs with a *N*_50_ value of 17.27 kb. Default parameters were used for all software unless otherwise specified.

The complete genome was annotated with Prokka v1.12 software (17), RAST (18), and BlastKOALA v2.1 (19), which predicted a total of 3,549 coding DNA sequences, 1 rRNA operon, and 53 tRNA genes. This allowed the identification of genes involved in dissimilatory nitrate reduction to ammonium, denitrification, aerobic respiration control protein ArcA, arsenate reductase, and resistance to heavy metals (Cu, Co, Zn, Cd, As, and Cr). Regarding the plasmid, 16 coding DNA sequences have been annotated and multiple hypothetical proteins detected.

The comparative analysis of the genome of *Brevundimonas* sp. strain T2.26MG-97 with those from other microbial subsurface isolates should provide insights on the mechanisms used by microorganisms to inhabit deep terrestrial anaerobic environments under oligotrophic conditions and in the absence of light.

### Data availability.

Reads have been deposited at DDBJ/ENA/GenBank under the accession numbers ERR2864313, ERR2864314, and ERR2864315. The complete genome sequences and annotations have been deposited under the accession numbers UXHF00000000 for the chromosome and UXHD00000000 for the plasmid, and they can be found under the BioProject accession number PRJEB29440. The versions described in this paper are the first versions, UXHF01000000 and UXHD01000000.
